# Outpatient clinics treating substance use disorders in Northwest Russia and Northern Norway: a descriptive comparative study

**DOI:** 10.1080/22423982.2017.1411733

**Published:** 2017-12-15

**Authors:** Helene Marie Dahl, Grigory Rezvyy, Anatoly Bogdanov, Terje Øiesvold

**Affiliations:** ^a^ Institute of Clinical Medicine, Faculty of Health Sciences, University of Tromsø, the Arctic University of Norway, Tromsø, Norway; ^b^ Division of general psychiatry, Nordland Hospital, Bodø, Norway; ^c^ Outpatient clinic of psychiatry, Kirkenes Hospital, Kirkenes, Norway; ^d^ Department of Psychiatry and Addictive Disorder, Arkhangelsk Clinical Psychiatric Hospital, Arkhangelsk region, Russia

**Keywords:** Alcohol use disorders, drug use disorders, health services, Barents region, International Classification of Mental Health Care, the European Service Mapping Schedule

## Abstract

Both in Norway and Russia a considerable portion of the population have substance use disorders. However, the knowledge about outpatient services treating substance use disorders in Norway and Russia is limited. This study will describe and compare outpatient clinics treating substance use disorders in Arkhangelsk in Northwest Russia and in Bodø and Tromsø in Northern Norway on availability, accessibility and treated prevalence (patients treated in one year). The managers (N=3) of the outpatient clinics (N=3) were interviewed with the European Service Mapping Schedule (ESMS) and the International Classification of Mental Health Care (ICMHC). The interviews were supplemented by e-mail and phone calls. The treatment in Arkhangelsk was mainly biologically oriented (medical), while a greater variety of methods was available in Bodø and Tromsø. The clinic in Russia was a drop-in clinic, while in Norway patients needed a referral to get an appointment in the clinic. Patients treated in Arkhangelsk (treated prevalence) was 1662, while in Bodø it was 233 and in Tromsø 220. The present study revealed great differences between the clinics involved in accessibility, availability and treated prevalence. Cultural traditions and budgeting of the mental health care system could explain some of the findings.

## Introduction

Both in Norway and Russia a considerable portion of the population is reported to have substance use disorders [1,2]. In Norway 12% of the men and 4% of the women (>15) were registered with an alcohol use disorder in 2010 [1], while in Russia the numbers for alcohol use disorders in 2010 were 31% for men and 6.2% for women [2]. The unlawfulness in both countries of illegal drugs makes it difficult to obtain exact information on this, but in a population survey in Norway in 2015 about 4.2% (aged 16–64 years) reported use of cannabis during the last 12 months, which is the dominant illegal drug in Norway [3]. Also, 64% of these were men [4]. In Russia the dominant illegal drugs are heroin and methamphetamine, and between 2% and 4% are regular drug users [5].

The mental health services in Norway are more differentiated and decentralised and more based on outpatient services than the Russian mental health services, which are largely hospital based as shown by Rezvyy et al. [6]. Among other known differences between Russia and Norway is the legislation concerning treatment for drug abuse as the government in Russia does not allow the use of substitution therapy for drug users [7].

Since the mid-1970s, services treating substance use disorders in Russia have been developed as a speciality separate from psychiatry, termed “narcology” [8], although they are still associated with the psychiatric services. Citizens can go to other physicians (usually neurologists) who are assigned responsibility for psychiatric patients [6]. Narcological services also serve people anonymously if paid for by the patient [9]. Patients in need for specialised services in Russia may establish a direct contact with this service. In contrast, entry to official mental health services in Norway is made via the general practitioners [6]. In Norway, patients with substance use disorders are referred to specialised services in the psychiatric health care system. The Regional Health Authorities carry the responsibility for cross-professional specialised treatment through a reform from 2004 which transferred the responsibility from the social services to the health care system [10]. Additionally, there are several private services in Norway, some of which have an arrangement with the Regional Health Authorities for expense refunds [11].

The aim of our study was to describe for the first time the specialised services treating substance use disorders in North West Russia (Arkhangelsk) and in Northern Norway (Tromsø and Bodø) thoroughly. Special emphasis will be given to availability and accessibility as well as treated prevalence. The purpose of this is to strengthen the foundation for cooperation in the mental health area between countries within the Barents region with health systems that differ in many ways.

## Materials and methods

### Design and data collection

This study was a multisite, cross-sectional descriptive study. Two standardised questioners, the European Service Mapping Schedule (ESMS) [12] and the International Classification of Mental Health Care (ICMHC) [13], were used. Treated prevalence, which describes the number of patients treated in the different clinics during 1 year (November 2010–November 2011) was collected. The data from the two clinics in Northern Norway (Tromsø and Bodø) were collected in December 2011 by the first (HMD) and second (GR) authors of this paper. GR (bilingual) also collected the data from the clinic in Arkhangelsk, North West Russia (February 2012). One manager of each clinic (N=3) was, after verbal consent was given, interviewed for about 90 min at his/her office and by phone or e-mail additionally. For the clinic in Arkhangelsk some data were estimated by the manager of the clinic due to lack of a registration system: average age of the patients was estimated based on a general impression in the clinic, and treated prevalence was estimated based on number of consultations registered through the year divided by average number of consultations for each patient. The data collected was on an institutional level.

### The questionnaires

The ESMS is designed for description of the package of mental health services providing care for the population in a catchment area [12]. The ESMS consists of questions that are related to the catchment area and its population, the service’s function (e.g. outpatient, continuing care, non-mobile), work setting and availability and the level of service use by the population. A supplemental section to elaborate further details is also included. The supplementary section was not used in this study as the information it provided was covered to a large extent by the second questionnaire, namely the ICMHC.

The ICMHC describes the availability of programmes and treatment methods within the clinics and classifies entities into “modules of care”, and subsequently in “modalities”. A module is framed by employees from one or several services that together are responsible for the treatment of the patients, and all employees on the modules’ pay list are considered a part of the module. Questions in ICMHC relate to the module’s main objectives of care (such as observation, support, assessment and rehabilitation), opening hours, full-time employees and type of service (such as inpatient, outpatient, mobile and non-mobile). Information related to the patients on a group level (such as age, diagnoses, sex and overall aim of treatment) is also described [13]. Furthermore, interventions and procedures to obtain specific objectives are classified into 10 modalities, rated (by the interviewers) from 0, which describe no systematic activity in the particular modality to obtain the objectives, to 3, which describes a high level of systematic activity in the modality to obtain the objectives. The objectives that are classified concern the relationship between clinic and patient, assessments and interventions concerning health and everyday problems, the patient’s relationship to family and significant others and coordination of care. To obtain a high rating certain criteria have to be fulfilled, such as availability of specialised services [13].

### Calculation

Calculations were done to find population density in the catchment area, percentage of the population in the city where the clinic was located, number of treatment staff per 1,000 patients, number of people in the catchment area per treatment staff member in the clinic, percentage of somatic treatment staff versus other staff members in the clinics, treated prevalence per treatment staff (the number of patients treated by one staff member) and treated prevalence (the number of treated patients) per 1,000 persons living in the catchment area. The same procedure was emphasised for all the three clinics in the study.

## Results

### The catchment areas

The clinic in Arkhangelsk (Russia) covered Arkhangelsk county, the clinic in Tromsø covered Troms and Finnmark counties and the clinic in Bodø (Norway) covered Bodø with nine neighbouring municipalities (Table 1). Compared to the Norwegian clinics (Tromsø and Bodø together), the clinic in Arkhangelsk had an area to cover that was eight and a half times larger, and a population about four times bigger to serve [14,15].

**Table 1.** Size of the catchment areas and population.

	Arkhangelsk	Bodø	Tromsø
Area (km^2^)	590,000	11,000	58,000
Population	1,300,000	77,400	240,000^a^
Population/km^2^	2	7	4
People living in the city where the clinic was located (%)	360,000 (28)	48,000 (62)	69,000 (30)

^a^75,700 live in Finnmark.

### Availability and accessibility

Human resources in the clinics are presented in Table 2. Concerning treatment staff per population in the catchment area, there lived approximately 80,000 persons in the area of Arkhangelsk per treatment staff member in the clinic compared to 13,600 in Bodø and 30,400 in Tromsø. The number of treatment staff per patient in the Russian clinic was about one-third of that in Norway. Concerning somatic treatment staff, however, the Norwegian clinics had none, while 62% of the staff in the Russian clinic was somatic treatment staff. The average time of consultation was 12 min in Arkhangelsk versus 60 min in each of the two Norwegian clinics in question.

**Table 2.** Availability of resources in the clinics and average consultation time.

	Arkhangelsk	Bodø	Tromsø
Total number of employees in the clinic	27.5	7.3	9.4
Narcologist	5.5	0	0
Psychiatrist	0.25	0.3	0.4
Psychologist	0	1.4^a^	3
Special psychologist	0	1	1
Psychiatric nurse	1: manager of nurses	1	1
Special advisor	0.5	2	2.5
Nurse	8.25	0	0
Injection nurse	2	0	0
Other^b^	3	1.6	1.5
Assistants^c^	7	0	0
Treatment staff^d^	16.5	5.7	7.9
Somatic treatment staff (%)^e^	10.25 (62)	0	0
Treatment staff members per 1,000 patients	10	24	36
Population in catchment area per treatment staff member	78,788	13,579	30,380
Average consultation time (min)	12	60	60

^a^One stationed on a regular basis in Bodø prison. ^b^Manager, secretary. ^c^Mainly looking after the patients in detoxification, but also involved in other tasks, like assisting other personnel and cleaning. ^d^All employees except assistants and “Other”. ^e^Nurses and injection nurses.

Based on the data collected by the ICMHC, the modules were described: assessment of the patients’ medical situation and treatment of their substance use were the main objectives of care in all three clinics. In both the Norwegian clinics support, psychotherapy and interventions concerning rehabilitation (in terms of resuming activities attached to, e.g. work and a social life) were the main activities, while in the Russian clinic rehabilitation was reported as a goal and not an activity. All clinics had as their main objective for treatment either abstinence or reduced use of substances, depending on the patients’ goal for the treatment. The goal was set in dialogue with the patient concerned. The main diagnosis in the Russian clinic was alcohol related (about 80%). In both the Norwegian clinics the main diagnosis was related to either drugs or alcohol, with drug use disorders being as frequent as alcohol use disorders. Only the Russian clinic offered detoxification, and treatment by a medical doctor specialised in substance use disorders (narcologist), and the patients were either registered for treatment or received unregistered treatment (appearing anonymous) if they paid for it.

Modality rating based on information collected by ICMHC (Table 3): modality 1 rates the activities needed to involve and keep the patients involved in their treatment [13]. The clinic in Arkhangelsk did not have routines for maintaining the relationship with the patient, while both the Norwegian clinics contacted the patient routinely via letter and phone calls, and if necessary, but not on a regular basis, went on a home visit. Modality 2, which rates the activities needed to make a plan for treatment and monitor its progress, was the only modality where all the clinics scored the same, and highest, rating. However, in the Norwegian clinics screening instruments were used on a regular basis to screen the mental health of the patients, while in the Russian clinic, they had a psychologist for assessing the mental health of the patient. Concerning somatic health, all patients were checked routinely in Arkhangelsk, while in both the Norwegian clinics the therapist assessed whether the patient needed a somatic examination. In Arkhangelsk the coordination of services (modality 3) was limited to letters and a phone call from the clinic to another service “now and then”. In Norway the coordination of services was on a regular basis, with phone calls, letters and sometimes meetings with the staff from the various services involved in the case of the patient. In modality 4, which rates the activities needed to provide general health care, the possibility to take care of both somatic and psychological health and having nurses, who, among other things, distributed medication, on their pay list, were the criteria for being rated. In the Norwegian clinics there were no nurses, and they were rated 0. The Russian clinic met the criteria due to several nurses on their pay list, as well as a psychiatrist and medical doctors (narcologists) who had a therapeutic role in the clinic, and so was rated 2. Modality 5, rating the level of support given to the patients in their activities of daily living, like washing, dressing, cleaning, cooking and shopping, was the only one in which none of the clinics had activity. Arkhangelsk and Tromsø were rated at the same level, level 2, in modality 6, concerning “psychopharmacological and other somatic interventions”, due to the possibility in both clinics to monitor the prescription of drugs closely and use specific techniques like laboratory tests. This may suggest similarity between the two clinics in psychopharmacological interventions, which was not the case. Psychopharmacological interventions (sodium chloride, or C vitamins and vitamins from group B, and benzodiazepines as tranquillizers) represented the main treatment in Arkhangelsk, which also offered detoxification. In Tromsø psychotherapy was the main treatment, and detoxification was not available. Bodø scored the lowest as the psychiatrist was in a training programme, and the prescription of drugs was done in consultation with the patients’ general practitioner. The psychiatrist in Tromsø was therefore at a higher level of specialisation than the psychiatrist in Bodø and monitored the prescription of drugs by laboratory tests, e.g. determining serum levels. Concerning psychological interventions (modality 7), which are mainly aimed at helping the patients to perceive and understand their thoughts and behaviour [13], Arkhangelsk had a psychotherapist who worked with rehabilitation in terms of giving the clients support. Well-defined psychotherapeutic interventions were developed only to a small extent. The clinics in Bodø and Tromsø employed cognitive therapy, psychodynamic methods and the method “Motivational Interviewing”. Additionally, Tromsø had several other methods and programmes. In modality 8 (interventions carried out to help people cope with and manage impairments and personal disabilities) and 9 (interventions aimed at helping and teaching individuals in how to use their days in a worthwhile way) the Russian clinic was assessed as having no activity. In both 8 and 9, the clinic in Bodø assessed the need and contacted other services if the need was present, as was also the case for the clinic in Tromsø in modality 9. In modality 8 the employees in the clinic in Tromsø had the possibility to provide interventions themselves. The rating of 1 in modality 10 assigned to the clinic in Arkhangelsk is due to the giving of advice and supporting conversations with the patients’ families. In Bodø the activity was screening of family and social situation and information concerning services outside the clinic, while in Tromsø services were provided directly, such as network groups, meetings with the patient and his or her children, and meetings with other family members.

**Table 3.** Modality ratings according to ICMHC.

Modality ratings (0–3)	Arkhangelsk	Bodø	Tromsø
1. Establishing and maintaining professional relationships	1	2	2
2. Problem and functional assessment	3	3	3
3. Care coordination	1	3	3
4. General health care	2	0	0
5. Taking over activities of daily living	0	0	0
6. Psychopharmacological and other somatic interventions^a^	2	1	2
7. Psychological interventions	1	2	3
8. Interventions aimed at helping to cope with disabilities^b^	0	1	2
9. Interventions related to daily activities	0	1	1
10. Interventions aimed at family, relatives and others	1	1	2

^a^Psychopharmacologial and other somatic interventions. ^b^Interventions aimed at managing disabilities, both physically and mentally.

As measured by the ESMS, all clinics provided continuing care with high intensity and they were non-mobile. Opening hours in Arkhangelsk were 24 h a day, 7 days a week, and between 8.00 am and 8.00 pm all services were available. From 8.00 pm to 8.00 am the professional resources still were available, but at a lower activity level. During these hours patients were encouraged to contact the medical emergency service. In both the Norwegian clinics the opening hours were 8.00 am to 3.30 pm 5 days a week (i.e. closed during weekends, as well as on public holidays). The clinic in Arkhangelsk was connected to a day centre and was a drop-in clinic. In contrast, a referral was required in Norway, either from the primary health care or another municipal contact, in order to get access to treatment. The referral was sent to an assessment team who decided whether there existed a need for treatment, and if so, what kind. The proportion of the entire population in the catchment area that lived in the city where the clinic was located was for the clinic in Bodø 62%, for Tromsø 32% and for the clinic in Arkhangelsk 28%.

### Treated prevalence

Tromsø scored the lowest in treated prevalence per population living in the catchment area, and Bodø the highest. The clinic in Arkhangelsk had more than twice the number of treated patients (101) per treatment staff member during 1 year as compared to Bodø (41) and Bodø had one third more than Tromsø, which had 28 (Table 4). There were a higher number of men in the Russian clinic than in the Norwegian clinics, and the patients were estimated to be from 2 to 7 years older in the Russian clinic than was found to be the case in both of the Norwegian clinics.

**Table 4.** One year treated prevalence from November 2010 to November 2011.

	Arkhangelsk	Bodø	Tromsø
Treated prevalence (TP)	1,662 (calculated)^a^	233	220
TP/treatment staff	101	41	28
Per 1,000 in catchment area	1.3	3	0.9
Average age last year	40–45^b^	39	38
Men/women	83/17	65/35	65/35

^a^Number of consultations registered through the year (24,931) divided by average number of consultations by each patient [15]. ^b^Estimated by the manager of the clinic.

## Discussion

The Russian clinic had fewer staff members per population in the catchment area (ratio 1:79,000) than the clinics in Tromsø (1:30,000) and Bodø (1:14,000). In general the community mental health services in Russia are concentrated in large cities and poorly developed in rural areas [6]. Arkhangelsk has vast rural areas, but only one outpatient clinic for treating substance use disorders. This may be a result of governmental prioritising of this type of treatment in areas with a high population density. Within Norway, Tromsø had an area to cover that was about 5.5 times larger than was the case for Bodø, with a population 3 times larger. This should imply a need for more staff in Tromsø, which was not the case, maybe due to different policy in what is giving priority, i.e. to what degree treating substance use disorders is given priority compared to other disciplines of mental health care.

Among the most striking differences found between the clinics was the duration of the consultations, which was on average 12 min for the clinic in Arkhangelsk and 60 min for both the Norwegian clinics. An explanation could be that the overall medical treatment (by pills and injections) in the Russian clinic may be less staff intensive than the psychotherapeutic treatment that was carried out in both the Norwegian clinics. Concerning the difference in treatment methods, this may be due to cultural differences [6]. There was also a difference between the Norwegian clinics, according to the number of patients treated by each staff member (Table 4) through 1 year (28 in Tromsø and 41 in Bodø). One explanation may be that the clinic in Tromsø offered a wider range of services to each patient and therefore spent more time and resources overall per patient than the clinic in Bodø (Table 3).

In contrast to the Norwegian clinics, diagnoses related to drug use were almost absent in the Russian clinic despite estimates that 2% of the adult population in Russia are opiate users, while the estimated global level is 0.4% [7]. It may be difficult, though, to provide an exact number, as being registered as a drug user has been stipulated as a definite barrier to enter treatment in Russian clinics [7,16]. Law enforcement agencies routinely check the lists, and the only way of being assured of confidentiality is by paying for anonymity [7]. It is likely that some, maybe many, of the drug abusers that attended the clinic were not able to pay for anonymity, which may have led to a situation of holding back information concerning their drug use. A lack of treatment methods may also affect the number of drug users that register. Methadone and buprenorphine maintenance programmes are illegal [7,17], and needle exchange programmes are limited [7], as well as effective social support programmes. However, there is some treatment offered if paid for by the patient. One is a hypnosis-based psychotherapy called “encoding” which is usually directed towards alcohol users, but is also used towards drug dependence [18], while another is the use of extended-release naltrexone that has proven effective both for alcohol dependence and drug dependence [19].

The present study found gender differences among the patients, and the difference was more emphasised in the Russian clinic (83% men) than in both the Norwegian clinics (65% men). According to Green et al. [20], alcohol appears to be a vital cultural and social part in the everyday lives of Russian men. The number for alcohol use disorders in Russia [2] corresponds to the distribution of men/women treated in the clinic.

The somatic focus in the Russian clinic versus the psychotherapeutic focus in the Norwegian clinics corresponds to findings in previous research [6,20]. In Arkhangelsk, 62% of the treatment staff had tasks involving medication and detoxification, and the majority of nurses were engaged in this. Although defined as therapeutic treatment in this study, the treatment in the Russian clinic was still medical (pills or injections) to a great extent when the patients met the narcologists, after being detoxified.

The Russian clinic was in many respects more accessible than the Norwegian ones due to the opening hours and the possibility of meeting a narcologist without a referral. In Norway it is necessary, after being referred by an official authority, to be authorised by yet another official authority deciding if there is a “right to treatment”. This bureaucracy may lower the accessibility as shown by Notley et al. [21].

Bodø had the highest treated prevalence per population, and also the smallest catchment area and therefore the smallest distance to the clinic for the majority of the patients. Distance to the clinic has been found to hinder utilisation of services [22–24] because of limited transportation abilities [22,25] and travel costs [26] in larger catchment areas. Despite the responsibility of the clinic in Arkhangelsk to treat people in a considerably larger area than the clinic in Tromsø, treated prevalence was higher than for the clinic in Tromsø. This may be explained both by opening hours and the number of patients treated per staff member in Arkhangelsk.

## Limitations and strengths

To interview the manager of the clinic about programmes available and intentions with treatment may generate a distorted picture as instructions and goals for the clinic may differ from actual practice. However, we do not think this has influenced our main findings, and the study is strengthened by the fact that the same person was conducting all the interviews. With this in mind we consider our findings to give a valid description of differences and similarities.

## Conclusions

The differences and similarities described in the study have revealed that the Northern Norwegian and the North West Russian outpatient clinics for treating substance use disorders differ to a large extent in both accessibility, availability and treated prevalence. We suggest some implications of the present research: policymakers should consider the establishment of several specialised outpatient clinics for treating substance use disorder, throughout the catchment area for the clinics in Arkhangelsk and Tromsø. The Russian policymakers should consider the prioritising of a greater variety of treatment programmes, as well as longer available time with the therapist and full anonymity for the clients without having to pay for it. The Norwegian policymakers should have accessibility in focus, especially the bureaucratic referral procedures. For clinicians, collaborative research like this may be useful in that it is always something to learn from each other concerning clinical practice.
